# Expression of a recombinant full-length LRP1B receptor in human non-small cell lung cancer cells confirms the postulated growth-suppressing function of this large LDL receptor family member

**DOI:** 10.18632/oncotarget.11897

**Published:** 2016-09-08

**Authors:** Arno G. Beer, Christoph Zenzmaier, Michael Schreinlechner, Jenny Haas, Martin F. Dietrich, Joachim Herz, Peter Marschang

**Affiliations:** ^1^ Department of Internal Medicine, Medical University of Innsbruck, Innsbruck, Austria; ^2^ University of Applied Sciences Tyrol, Innsbruck, Austria; ^3^ Department of Molecular Genetics, University of Texas Southwestern Medical Center, Dallas, TX, USA

**Keywords:** low-density lipoprotein receptor-related protein 1B, tumor suppressor, proliferation, overexpression, siRNA

## Abstract

Low-density lipoprotein (LDL) receptor-related protein 1B (LRP1B), a member of the LDL receptor family, is frequently inactivated in multiple malignancies including lung cancer. LRP1B is therefore considered as a putative tumor suppressor. Due to its large size (4599 amino acids), until now only minireceptors or receptor fragments have been successfully cloned. To assess the effect of LRP1B on the proliferation of non-small cell lung cancer cells, we constructed and expressed a transfection vector containing the 13.800 bp full-length murine *Lrp1b* cDNA using a PCR-based cloning strategy. Expression of LRP1B was analyzed by quantitative RT-PCR (qRT-PCR) using primers specific for human *LRP1B* or mouse *Lrp1b.* Effective expression of the full length receptor was demonstrated by the appearance of a single 600 kDa band on Western Blots of HEK 293 cells. Overexpression of *Lrp1b* in non-small cell lung cancer cells with low or absent endogenous LRP1B expression significantly reduced cellular proliferation compared to empty vector-transfected control cells. Conversely, in Calu-1 cells, which express higher endogenous levels of the receptor, siRNA-mediated LRP1B knockdown significantly enhanced cellular proliferation. Taken together, these findings demonstrate that, consistent with the postulated tumor suppressor function, overexpression of full-length *Lrp1b* leads to impaired cellular proliferation, while *LRP1B* knockdown has the opposite effect. The recombinant *Lrp1b* construct represents a valuable tool to unravel the largely unknown physiological role of LRP1B and its potential functions in cancer pathogenesis.

## INTRODUCTION

LRP1B is a member of the LDL receptor family of lipoprotein receptors, which are involved in different functions in the human body including cholesterol metabolism and atherosclerotic lesion formation. LRP1B was originally discovered during the study of lung cancer cell lines [1]. In nearly 50% of non-small cell lung cancer (NSCLC) cell lines, alterations of the *LRP1B* gene with homozygous deletions of exons or abnormal transcripts missing portions of the *LRP1B* sequence were observed. Therefore, LRP1B was postulated as a putative tumor suppressor. In subsequent studies, LRP1B was found to be inactivated in multiple malignancies, namely urothelial cancer, hepatobiliary tumors, esophageal carcinoma, cervix carcinoma, glioblastoma, oral squamous cell carcinoma, small B-cell lymphoma, acute lymphoblastic leukemia, gastric cancer, thyroid cancer, melanoma, ovarian cancer, renal cell cancer, and adrenocortical carcinoma [2–15]. Besides allelic loss of heterozygosity and inactive mRNA transcripts, DNA methylation of CpG islands has been described as mechanism leading to decreased *LRP1B* expression in various tumors [4, 8–11]. Recently, LRP1B was identified as integration site for hepatitis B virus and human papilloma virus presumably with impact on LRP1B expression [16, 17]. Taken together, these observations strongly suggest a role of LRP1B in tumorigenesis and strengthen the original hypothesis of the receptor serving as a tumor suppressor. Recently, we have characterized the expression of LRP1B in normal human tissues, which appears to be mostly restricted to brain, skeletal muscle, thyroid gland and testis. In addition, expression in smooth muscle cells of the arterial wall has been described [18].

LRP1B is one of the largest transmembrane receptors comprising 4599 amino acids encoded by an mRNA of 13800 base pairs. Similar to the homologous LRP1 receptor, LRP1B contains four ligand binding domain regions separated by EGF precursor homology regions, a transmembrane segment and a cytoplasmic tail containing two NPxY motifs [1]. In contrast to the homologous LRP1, LRP1B is not cleaved by furin and therefore migrates as single polypeptide chain with an apparent molecular weight of 600 kD on SDS polyacrylamide gels [19].

To gain insight into the physiological functions of LRP1B, a knockout mouse model has been generated by replacing the transmembrane domain (exon 88) with a neomycin cassette, resulting in the absence of a membrane-inserted receptor. These mice were viable and fertile and did not show any obvious abnormalities, including no increased tumor rate [19]. However, when the *Lrp1b* gene was inactivated by more proximal deletions, no viable homozygous *Lrp1b* mutant animals were obtained, strongly suggesting a crucial role for the extracellular domain in normal development [20].

To further characterize the physiological function of the receptor, several attempts have been made to construct a recombinant LRP1B receptor. However, due to the enormous size of the polypeptide chain, only minireceptors comprising a part of the LRP1B sequence (ligand binding domain region IV, transmembrane segment and intracellular tail) and soluble ligand binding ectodomains have been constructed [19, 21]. In the present study we used a PCR-based strategy to construct a recombinant full-length *Lrp1b* expression vector. This recombinant receptor was then introduced into human cells lacking endogenous LRP1B and cellular proliferation was analyzed. To exclude artifacts caused by overexpression, control experiments using siRNA to silence LRP1B expression were performed.

## RESULTS

### Amplification and subcloning of N-terminal, middle and C-terminal *mLrp1b* fragments

**Figure 1:**
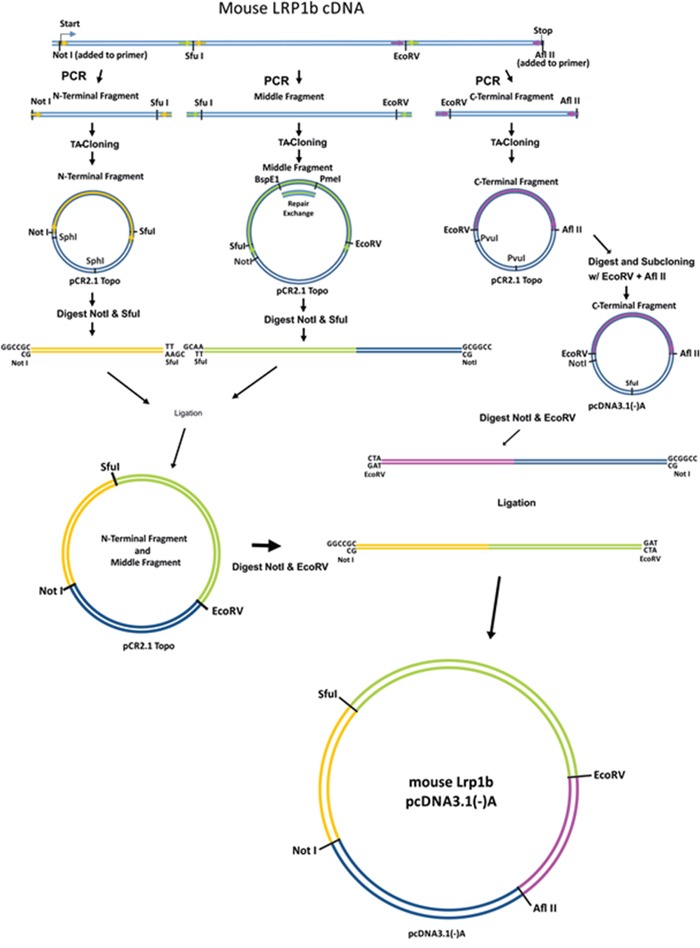
Cloning strategy to construct a full-length *Lrp1b* receptor The murine *Lrp1b* m-RNA was divided into three fragments separated by unique restriction sites and amplified from mouse brain cDNA using specific primers containing the indicated unique restriction sites. After subcloning in a TA vector and repair of a GC rich sequence within the middle fragment, the fragments were joined and cloned into the pcDNA3.1 (-)/myc-His A expression vector. The myc and HIS tag were excised during the cloning process.

Due to the enormous size of the *mLrp1b* cDNA (13.8 kb, Genebank NM_053011), the coding sequence was divided into three parts (N-terminal (3810 bp), middle (5970 bp) and C-terminal (4020 bp) fragments) and amplified separately from mouse brain cDNA using specific primers (Figure 1). To ensure efficient transcription, a Kozak consensus sequence was included preceding the start codon within the N-terminal fragment. The integrity of the sequences was confirmed by restriction enzyme digestion and complete sequencing. Even with polymerases containing proof reading enzymes, single base substitutions cannot be avoided in these large amplified DNA segments. Therefore, in several instances multiple amplified clones had to be analyzed to obtain sequences without non-conservative base exchanges. We found a particular high rate of base substitutions in a 2213 bp GC-rich region within the middle fragment flanked by two unique restriction sites (BspEI and PmeI). Therefore, this region was amplified separately and then reinserted into the middle fragment.

### Construction and expression of the full-length *mLrp1b* expression vector

First, the C-terminal fragment was cut out of the TOPO vector using EcoRV(5′) and AflII(3′) followed by degradation of the vector backbone with PvuI, allowing the separation of the insert from the vector of similar size by preparative agarose gel electrophoresis. The C-terminal fragment was then cloned into the mammalian expression vector pcDNA3.1(-)/myc-HisA using the same unique restriction enzyme sites. To avoid a three fragment ligation with large fragments, the N-terminal and middle fragment were subcloned into the pCR2.1 Topo vector. To this end, the vector containing the N-terminal fragment was cut with SfuI and NotI. Also in this case, degradation of the vector backbone with SphI was necessary to allow the separation of the insert from the vector by preparative agarose gel electrophoresis. The N-terminal insert was subsequently ligated into the linearized (NotI and SfuI) middle-insert containing vector.

**Figure 2:**
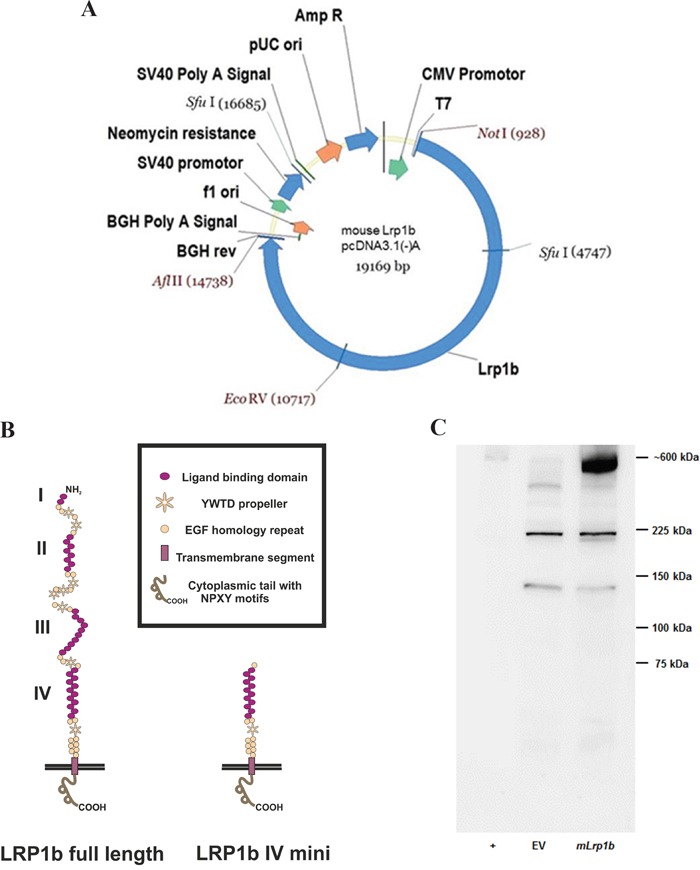
Expression of full-length *mLrp1b in HEK 293 cells* **A.** The pcDNA 3.1 mammalian expression vector containing the full length *mLrp1b* cDNA is shown with the unique restriction enzyme sites used for cloning. **B.** Comparison of the protein structure of the full length murine Lrp1b protein and the receptor containing only domain IV (i.e. minireceptor) **C.** HEK 293 cells were transfected with plasmids containing either full-length *mLrp1b* or empty vector (EV). Protein lysates were prepared and subjected to Western blotting using a polyclonal antibody directed against a C-terminal epitope. Crude membrane preparations from mouse brain served as positive control (+).

The last step was to cut the pcDNA3.1 vector containing the C-terminal fragment with EcoRV and NotI, preparing the joined N-terminal and middle fragment insert using the same enzymes, and ligating the two parts to obtain the full-length *Lrp1b* expression vector (Figure 2A). The integrity of the sequence was confirmed by sequencing of the whole insert using the sequencing primers shown in Supplementary Table S1 thereby confirming the absence of any non-conservative mutations. Due to the usage of the AflII site located between the HIS-tag and the BGH polyadenylation signal, the myc and HIS tag were excised from the vector backbone and the construct therefore codes for the full-length *Lrp1b* receptor without a C-terminal tag. A comparison of the protein structure of the full length murine Lrp1b protein and the previously established human LRP1B region IV minireceptor [19] is depicted in Figure 2B.

The full-length expression vector and the empty vector as negative control were linearized with NruI and subsequently transfected into HEK 293 cells. Transfected cells were selected by resistance to G418 and tested for effective expression by Western blotting with a C-terminal polyclonal antibody. As shown in Figure 2C, in HEK 293 cells transfected with the *mLrp1b* construct, a band at approximately 600 kD appeared, corresponding to a band with the same apparent molecular weight in the positive control lane (mouse brain lysate). The lower bands in the *mLrp1b*-transfected HEK 293 cells are non-specific, since they were also observed in the negative control (empty vector).

### Basal *LRP1B* expression in human non-small cell lung cancer cell lines

**Figure 3:**
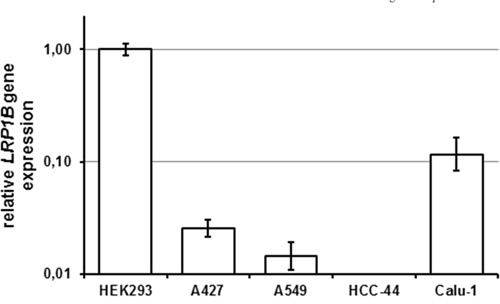
Endogenous *LRP1B* levels in non-small cell lung cancer cell lines Endogenous *LRP1B* mRNA levels in A427, A549, HCC-44 and Calu-1 cells were measured by quantitative PCR. Gene expression levels were normalized using the housekeeping gene *RPLP0* and are shown relative to HEK 293 cells. Values are depicted as mean and SEM and are representative for at least three independent experiments run in triplicates.

To identify cell lines lacking endogenous *LRP1B* expression, NSCLC cell lines were tested for the presence of *LRP1B* transcripts using a sensitive quantitative RT-PCR (qPCR) with primers specific for human *LRP1B*. HEK 293 cells, which have been previously shown to express endogenous *LRP1B* [22], served as positive control. As shown in Figure 3, two NSCLC cell lines (A427, A549) showed very low *LRP1B* expression. In another NSCLC cell line (HCC-44), no *LRP1B* transcripts could be detected with this very sensitive assay. In contrast, another NSCLC cell line (Calu-1) showed relatively high *LRP1B* expression (about 1/10 of HEK 293 cells).

### Expression of full-length *Lrp1b* inhibits proliferation of non-small cell lung cancer cells

**Figure 4:**
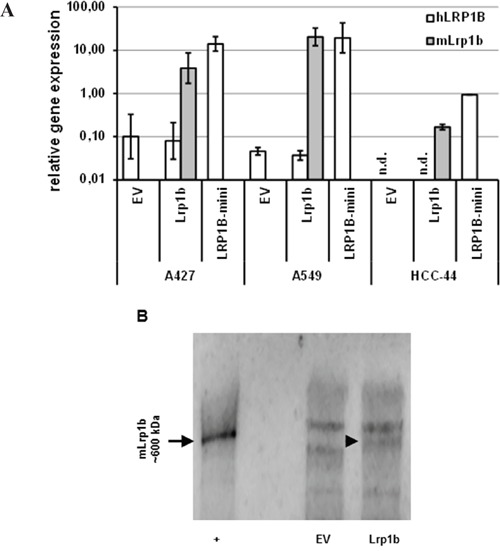
Effective expression of *Lrp1b* in non-small cell lung cancer cells **A.** A427, A549 and HCC-44 cells were stably transfected with plasmids containing either murine full-length *Lrp1b* or a human LRP1B minireceptor (LRP1B-mini). Empty vector (EV) transfected cells served as control. The level of expression of hLRPB (endogenous and hLRP1B minireceptor) and *mLrp1b (full-length mLrp1b)* were determined using primers specific for the human (*hLRP1B*) and mouse (*mLrp1b*) sequences, respectively. Gene expression levels were normalized using the housekeeping gene *RPLP0* and are shown relative to endogenous *LRP1B* levels of HEK 293 cells. Values are given as mean and SEM and are representative for at least three independent experiments run in triplicates. n.d. not detectable. **B.** Verification of full-length mLRP1B overexpression at protein levels in A427 cells. Western blot analysis of total cell lysates from Lrp1b transfected cells and empty vector control using a polyclonal rabbit-anti LRP1B antibody (recognizing both the human and mouse sequences). Crude membrane preparations from mouse brain served as positive control (+).

Given their very low endogenous *LRP1B* expression levels, the NSCLC cell lines A427, A549 and HCC-44 were used to investigate the effect of *Lrp1b* overexpression on cellular proliferation. Thus, the cell lines were stably transfected with full-length *Lrp1b* or a human LRP1B minireceptor. The expression of *mLrp1b* and *hLRP1B* transcripts in these transfected cells was measured by qPCR using primers specific for the mouse and human sequences, respectively. As shown in Figure 4A, *mLrp1b* and hLRP1B minireceptor in transfected cells was higher (A427, A549) or at least similar (HCC-44) compared to endogenous *LRP1B* levels in HEK 293 cells. Efficient expression of full-length *mLRP1B* protein was proven by immunoblotting using a polyclonal LRP1B-specific antibody (Figure 4B).

**Figure 5:**
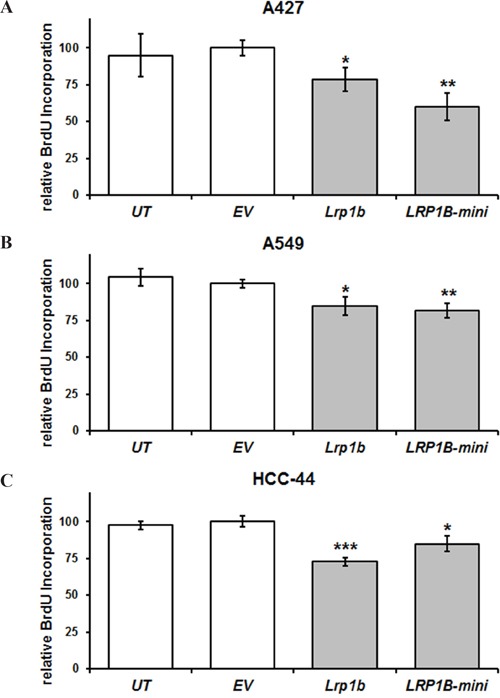
*Lrp1b* overexpression attenuates cellular proliferation of non-small cell lung cancer cell lines *in vitro* Relative quantification of DNA synthesis as determined by bromodeoxyuridine (BrdU) incorporation ELISA is shown for **A.** A427 **B.**, A549, and **C.** HCC-44 NSCLC cells stably transfected with plasmids containing either full-length *mLrp1b* or a hLRP1B minireceptor (LRP1B-mini). Untransfected (UT) and empty vector (EV) transfected cells served as controls. Values are given as mean and SEM and are representative for at least three independent experiments run in triplicates. Significance versus EV is indicated (* *P* < 0.05, ** *P* < 0.01, *** *P* < 0.001).

Subsequently, cellular proliferation of stably transfected cells was investigated. In agreement with its proposed tumor suppressor function, overexpression of full-length *mLrp1b* significantly reduced cellular proliferation of A427, A549 and HCC-44 cells in a BrdU incorporation assay. Stable transfection with the hLRP1B minireceptor resulted in comparable attenuation of proliferation (Figure 5).

To investigate if this anti-proliferative effect was specific for LRP1B or could be also mimicked by highly related proteins such as LRP1, a previously established full length LRP1 receptor [23] was employed. However, in contrast to full-length *mLrp1b* or the hLRP1B minireceptor, LRP1 overexpression did not significantly affect cellular proliferation of A549 (Supplementary Figure S1) and A427 cells (data not shown), respectively.

### siRNA-mediated LRP1B knockdown increases cellular proliferation of Calu-1 cells

To verify that the reduced proliferation observed in NSCLC cells expressing *mLrp1b* was a specific effect of *Lrp1b* and not due to side effects or overexpression artefacts (e.g. endoplasmatic reticulum stress (unfolded protein response) that is commonly induced by overexpression of highly-glycosylated proteins), siRNA-mediated *LRP1B* knockdown was applied. To this end, Calu-1 NSCLC cells were selected due to their relatively high endogenous *LRP1B* levels (Figure 3).

**Figure 6:**
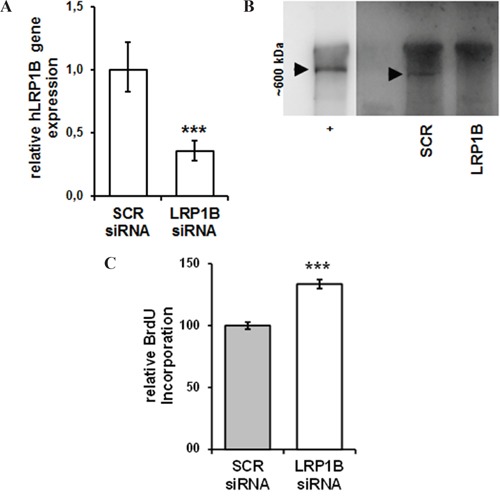
siRNA-mediated knockdown of LRP1B increases Calu-1 proliferation Calu-1 NSCLC cells were transfected with a pool of *LRP1B* specific siRNAs or non-target scrambled (SCR) control siRNAs. **A.** Effective knockdown of *LRP1B* is shown by quantitative PCR relative to Calu-1 cells treated with scrambled siRNA and normalized using the housekeeping gene *RPLP0*. **B.** Western blot analysis of total cell lysates using a polyclonal rabbit-anti LRP1B antibody. Crude membrane preparations from mouse brain served as positive control (+). **C.** Relative quantification of DNA synthesis as determined by bromodeoxyuridine (BrdU) incorporation ELISA. Values are given as mean and SEM and are representative for at least three independent experiments run in triplicates. Significance versus SCR is indicated (*** *P* < 0.001).

Transfection with *LRP1B*-specific siRNA resulted in an approximately 65% reduction of *LRP1B* mRNA levels compared with non-targeting control siRNA (Figure 6A) and effective knockdown was verified at protein levels (Figure 6B). In contrast to the attenuated cellular proliferation of NSCLC cells observed after *Lrp1b* overexpression, *LRP1B* knockdown significantly enhanced the proliferation of Calu-1 cells (Figure 6C).

### Expression of full-length *Lrp1b* increases p21^CIP1^ levels and Stat3 phosphorylation and reduces Src phosphorylation in A427 cells

To gain first insights into the molecular mechanism underlying the growth attenuation caused by *Lrp1b* overexpression, the impact of previously identified potential LRP1B ligands on cellular proliferation was investigated. Therefore, A427 cells stably transfected with the full-length *mLrp1b* or empty vector control were stimulated with various concentrations of very low-density lipoprotein (VLDL) or fibrinogen, putative ligands that have been shown to bind to the minireceptor and/or soluble ectodomains previously [22]. However, while VLDL did not affect proliferation rate and fibrinogen dose-dependently enhanced proliferation of both *Lrp1b* overexpressing and control cells, *Lrp1b* overexpression constantly attenuated proliferation rate by 30-40% irrespective of VLDL or fibrinogen supplementation at all concentrations tested (Supplementary Figure S2).

**Figure 7:**
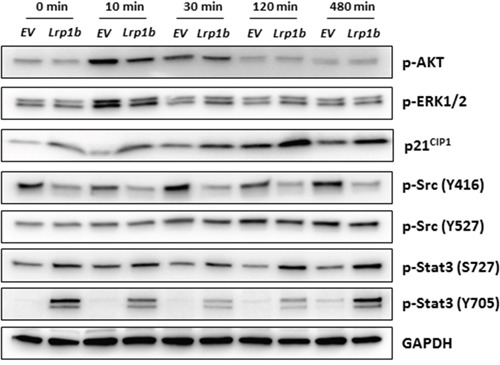
Effect of Lrp1b overexpression on intracellular signalling pathways A427 cells stably transfected with plasmids containing either murine full-length *Lrp1b* or empty vector (EV) control were serum-starved overnight and subsequently stimulated with 10% fetal calf serum for different time intervals. Thereafter, total protein lysates were analyzed by Western blotting using the indicated antibodies. GAPDH served as loading control. Images are representative of three independent experiments.

Additionally, several intracellular signaling pathways that potentially control cell proliferation were analyzed in A427 cells by Western blot analysis using (phosphorylation) specific antibodies (Figure 7). *mLrp1b* overexpression did not significantly affect activation of protein kinase B (AKT) and mitogen-activated protein kinase (MAPK) signaling in response to serum stimulation as determined by levels of phosphorylated AKT and ERK1/2, respectively. However, protein levels of the cyclin-dependent kinase inhibitor 1 (CDKN1A/p21^CIP1^) were enhanced in cells overexpressing *mLrp1b* whereas phosphorylation of proto-oncogene tyrosine-protein kinase Src at residue Tyr416 was attenuated. Moreover, phosphorylation of the transcription factor signal transducer and activator of transcription 3 (Stat3) was increased in A427 transfected with the full-length *mLrp1b* vector compared with empty vector control cells (Figure 7).

## DISCUSSION

Using a PCR-based cloning strategy, we were able to construct a mammalian expression vector encoding the entire murine *Lrp1b* mRNA (13800 base pairs). The construct was introduced by transfection into human cell lines (HEK 293 and NSCLC cell lines). Using a sensitive qPCR method to detect and discriminate *hLRP1B* and *mLrp1b* mRNA sequences, efficient expression of the full-length mLrp1b construct in these cells was proven. Moreover, mLRP1B protein expression was demonstrated by the appearance of an approximately 600 kDa band on Western blots using a specific affinity-purified antibody. As described previously, LRP1B is expressed as a single polypeptide chain and, unlike the homologous LRP1, does not undergo proteolytic cleavage by furin [19]. Consistently, overexpression of *mLrp1b* resulted in a single band in Western blot analysis without any additional bands that would indicate proteolytic cleavage (Figure 2C). To the best of our knowledge, this represents the largest transmembrane receptor expressed successfully in recombinant form in mammalian cells.

Previous studies that tried to identify extracellular ligands of LRP1B by using LRP1B minireceptors and soluble ectodomain constructs revealed a variety of potential binding partners, including VLDL and fibrinogen, [21, 22, 24]. In addition, several well-known or postulated molecular chaperones were identified by affinity chromatography with these ectodomains, highlighting the important role of chaperones in the biosynthesis and correct folding of this complex receptor [19, 25]. When an LRP1B minireceptor was introduced into mammalian cells, a slower endocytosis rate compared to analogous minireceptor constructs derived from LRP1 has been reported [21, 26]. In addition, a slower internalization rate of uPA/PAI- complexes and a decreased migration rate were described in cells expressing the LRP1B minireceptor compared to cells expressing a homologous LRP1 construct [24]. While minireceptors are very useful tools in identifying ligands or mapping binding sites, they may not necessarily share all biological functions with the full –length receptor molecules [27, 28]. Notably, in the present study the previously identified potential LRP1B ligands VLDL and fibrinogen did not alter the growth-suppressing function of the receptor in A427 cells.

In agreement with the *LRP1B* inactivation in many malignancies and the thus postulated putative tumor suppressor function [1–15], we identified NSCLC cell lines with very low (A427, A549) or absent (HCC-44) endogenous *LRP1B* expression. In contrast, in another NSCLC cell line, Calu-1 a relatively high endogenous *LRP1B* expression was observed.

In NSCLC cells with low/absent endogenous *LRP1B* levels overexpression of full-length *mLrp1b* or a LRP1B minireceptor resulted in marked inhibition of cellular proliferation compared to empty vector transfected cells, strongly supporting the proposed tumor suppressor function. Moreover, these findings are in line with previous results demonstrating that a LRP1B minireceptor suppressed anchorage-independent growth of *LRP1B*-deficient H4 neuroglioma cells [29]. The growth-suppressing function of LRP1B was further corroborated by the fact that siRNA-mediated silencing of endogenous *LRP1B* in Calu-1 cells significantly increased cellular proliferation. Similarly, knockdown of *LRP1B* significantly promoted anchorage-independent growth in HEK 293 cells and renal cancer cells 127 [14].

The molecular mechanisms underlying the growth-suppressing function of LRP1B remain elusive. Our findings indicate that this anti-proliferative activity was not affected by addition of the potential receptor ligands VLDL or fibrinogen. Moreover, *Lrp1b* overexpression appeared not to affect mitogen-stimulated intracellular AKT and MAPK/ERK signaling. However, *Lrp1b* overexpression increased levels of p21^CIP1^, a cyclin-dependent kinase inhibitor that blocks cell cycle progression from G1 to S phase [30]. Moreover, phosphorylation of Src at residue Tyr416 was attenuated in *Lrp1b* expressing A427 cells. Src represents an important proto-oncogene that promotes cellular proliferation. Scr activation has been documented in more than 50% of tumors derived from colon, liver, lung, breast, and pancreas and [31]. Of note, whereas overexpression of *Lrp1b* appeared to inactivate Src previous findings demonstrated that Src can be activated by LRP1 [32].

Given that constitutive activation of Stat3 is a frequent aberrancy in cancer cells that is supposed to directly contribute to tumorigenesis, the observed activation of Stat3 in A427 cells overexpressing the proposed tumor suppressor *Lrp1b* appears contradictory. However, newer studies indicated that Stat3 exerts also tumor suppressor effects under specific conditions. As reviewed recently, Stat3 can function either as an oncoprotein or a tumor suppressor in the same cell type, depending on the specific genetic background or presence/absence of specific coexisting biochemical defects [33].

Future research will have to address these mechanisms in more detail and is required to determine whether the growth-suppressing effect of LRP1B extends from the inhibition of tumor cell growth to a possible role of LRP1B in other cells, as e.g. the formation of atherosclerotic lesions.

## MATERIALS AND METHODS

### Cloning of full-length murine *Lrp1b*

Total RNA was prepared from mouse brain (C57black6/J) using RNAStat (Amsbio) and 1μg transcribed into cDNA using Oligo dT12-18 (Invitrogen) and SuperScript® II Reverse Transcriptase (Clontech) as suggested by the manufacturer. Preparative PCR was performed with Takara-brand LA PCR Kit, Version 2.1 (Clontech) according to the manufacturer's instructions.

Restriction enzymes were purchased either from NEB, Fermentas or Roche and used as recommended by the manufacturer (with 5-fold amount of enzyme for cleaving nonlinear DNA).

Plasmids were purchased from Invitrogen (pcDNA3.1™(-)/myc-His A and pCR2.1 Topo, the latter being part of the TA Cloning Kit). DNA concentrations were determined photometrically. For agarose gel electrophoresis SeaKem® LE Agarose (Lonza) was used at appropriate concentrations containing GelRed™ Nucleic Acid Gel Stain (Biotium) for analytic gels, and SeaKem® GTG Agarose (Lonza) for preparative gels, respectively.

TaKaRa DNA Ligation Kit Long (Clontech) and DNA ligation kit version 2 (Takara) were used for ligation reactions as suggested including appropriate negative and positive controls. TaKaRa DNA Ligation Kit Long was used for ligations involving fragments longer then 10kB (Ligation for 15h at 16°C). Subcloning of PCR fragments into the pCR2.1 Topo TA vector was performed as suggested by the manufacturer.

For transformation reactions XL10-Gold Ultracompetent Cells purchased from Agilent technologies (for longer fragments) or Library efficient DH5a competent cells (Gibco) were used. Selection was done on LB agar plates containing 100mg/l Ampicillin (Sigma). Clones were picked and grown in LB medium containing 100mg/l ampicillin overnight. Plasmid DNA was prepared using QIAprep Spin Miniprep Kit (Qiagen).

Large scale plasmid preparation was done using Qiagen Plasmid Maxi Kit (Qiagen). Digestion was performed as suggested by the manufacturer. For preparative digestions, an aliquot of the reaction was removed after 3 hours and subjected to gel electrophoresis for verification of complete digestion.

Sequences of all used primers are given in Supplementary Table S1.

### Cell lines and cell culture

Human embryonal kidney (HEK) 293 cells and Calu-1 NSCLC cells were purchased from the American Type Culture Collection (ATCC) and cultured in Dulbecco's modified Eagle medium DMEM (Lonza) supplemented with 10 % fetal calf serum (FCS) (Sigma Aldrich), 4mM glutamine (PAA Laboratories) and 1% penicillin/streptomycin (PAA Laboratories). NSCLC cell lines A427, A549 and HCC-44 were purchased from the German Collection of Microorganisms and Cell Cultures (DSMZ) and cultured in RPMI 1640 (Lonza) supplemented with 10 % fetal calf serum (FCS), 4mM glutamine and 1% penicillin/streptomycin.

### Transfection of HEK 293 cells with the full-length *mLrp1b* expression vector

HEK 293 cells were grown to 80-90% confluency. After changing the medium (DMEM) to antibiotic free medium, FuGENE 6 transfection reagent (Promega) was used according to the manufacturer's instructions. Briefly, for a 25 cm^2^ flask with 2 ml medium, 100μl serum free medium plus 6μl Fugene and 2μg of linearized plasmid were used for the transfection mix. After 6 hours the transfected cells were selected and maintained in the presence of G418 (Gibco) at 0.4 mg/ml.

### Overexpression and knockdown of LRP1B in non-small lung cancer cells

A427, A549 amd HCC-44 cells were stably transfected with the generated full-length *Lrp1b* vector, a plasmid coding for a human LRP1B region IV minireceptor [19] or an empty vector as control, respectively, using FuGENE 6 as described above.

To knock down endogenous LRP1B expression, Calu-1 cells were transfected with 50 nM SMARTpool: ON-TARGETplus LRP1B siRNA (Dharmacon) or scrambled (SCR) control siRNA (ON-TARGETplus non-targeting pool; Dharmacon) using DharmaFect 1 transfection reagent (Dharmacon) according to manufacturer's instructions.

### RNA isolation, reverse transcription and quantitative real-time PCR

Total RNA was extracted from whole cells using RNeasy Mini Kit (Qiagen) according to manufacturer's instructions and reverse-transcribed with M-MLV reverse transcriptase (Promega). Quantitative PCR (qPCR) was performed using the SsoFast™ EvaGreen® Supermix (BioRad) and the CFX96 PCR System (BioRad) with CFX96 Manager software on a Windows 7 platform according to manufacturer's instructions. The specificity of PCR products was confirmed by melting curve analysis. cDNA concentrations were normalized to the internal standard ribosomal protein, large, P0 (*RPLP0*), a moderate copy number housekeeping gene not regulated under the experimental conditions used. Fold change in gene expression was determined using the mathematical model ratio 2^−ΔΔCT^ [34]. Primers were designed using Primer3Plus [35] to allow specific quantification and differentiation of human *LRP1B* (endogenous receptor, LRP1B minireceptor) and mouse *Lrp1b* (full-length recombinant Lrp1b receptor) sequences. Sequences of all used primers are given in Supplementary Table S1.

### Western blot analysis

Total cell extracts were prepared as described previously [36] and were separated with 4-15% reducing SDS gels followed by immunoblotting with an affinity-purified, polyclonal anti-LRP1B antibody raised against the 13 C-terminal amino acids (identical in human LRP1B and mouse Lrp1b) as previously described [19]. Crude membrane preparations from mouse brain were prepared as described [19] and used as positive control.

### Proliferation assay

Relative quantification of DNA synthesis was determined by bromodeoxyuridine (BrdU) incorporation as described previously [37]. Briefly, stably transfected NSCLC cells or Calu-1 cells 72 h post-transfection with siRNA were incubated at a density of 5000/well in 96 well plates in culture medium containing 1% FCS. Proliferation rate after 72 h was analyzed by the Cell Proliferation ELISA BrdU Kit (Roche Applied Science) according to manufacturer's instructions.

### Identification of intracellular signaling pathways

A427 cells stably transfected with plasmids containing either murine full-length *Lrp1b* or empty vector (EV) control were serum-starved overnight and subsequently stimulated with 10% fetal calf serum. Total cell extracts from these cells were analyzed using the following antibodies: Phospho-Akt (Cell Signaling Technology #9271); Phospho-p44/42 MAPK (Erk1/2; Cell Signaling Technology #9101); p21^CIP1^ (BD Pharmingen #556430); Phospho-Src (Tyr416; Cell Signaling Technology #2101); Phospho-Src (Tyr527; Cell Signaling Technology #2105); Phospho-Stat3 (Ser727; Cell Signaling Technology #9134); Phospho-Stat3 (Tyr705; Cell Signaling Technology Antibody #9131); GAPDH (Abcam #ab37187).

### Statistics

Numeric data are presented as mean ± SEM from at least three independent experiments. Statistical differences between treatments were calculated using Student's paired t-test.

## SUPPLEMENTARY MATERIALS FIGURES AND TABLE




