# Supplementary material for: Expression of a recombinant full-length LRP1B receptor in human non-small cell lung cancer cells confirms the postulated growth-suppressing function of this large LDL receptor family member
Source: Oncotarget. 2016 Sep 8;7(42):68721–33. doi: 10.18632/oncotarget.11897 (PMC5356585; doi:10.18632/oncotarget.11897)
Supplement: Supplementary file 1 [file oncotarget-07-68721-s001.pdf]

Expression of a recombinant full-length LRP1B receptor in human non-small cell lung cancer cells confirms the postulated growth-suppressing function of this large LDL receptor family member

SUPPLEMENTARY FIGURES AND TABLE

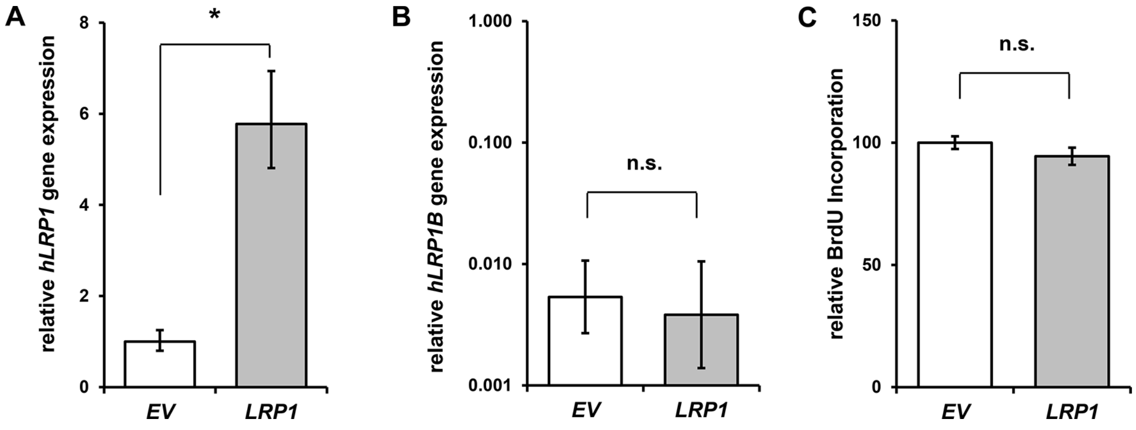

Supplementary Figure S1. *hLRP1* overexpression does not affect cellular proliferation of A549 non-small cell lung cancer cells *in vitro*. A549 cells were stably transfected with plasmids containing either human full-length *LRP1* or empty vector (EV) control. The expression levels of *hLRP1* and *hLRP1B* were determined by quantitative PCR and are shown relative to endogenous *hLRP1* (EV). **A.** Effective overexpression of hLRP1 in A549 cells did not affect **B.** hLRP1B expression levels, that were approximately 200 fold lower compared with endogenous LRP1 (note logarithmic scale in (B). **C.** Relative quantification of DNA synthesis as determined by BrdU incorporation ELISA demonstrated that *hLPR1* overexpression did not affect cellular proliferation of A549 cells. Values are given as mean and SEM and are representative for at least three independent experiments run in triplicates.

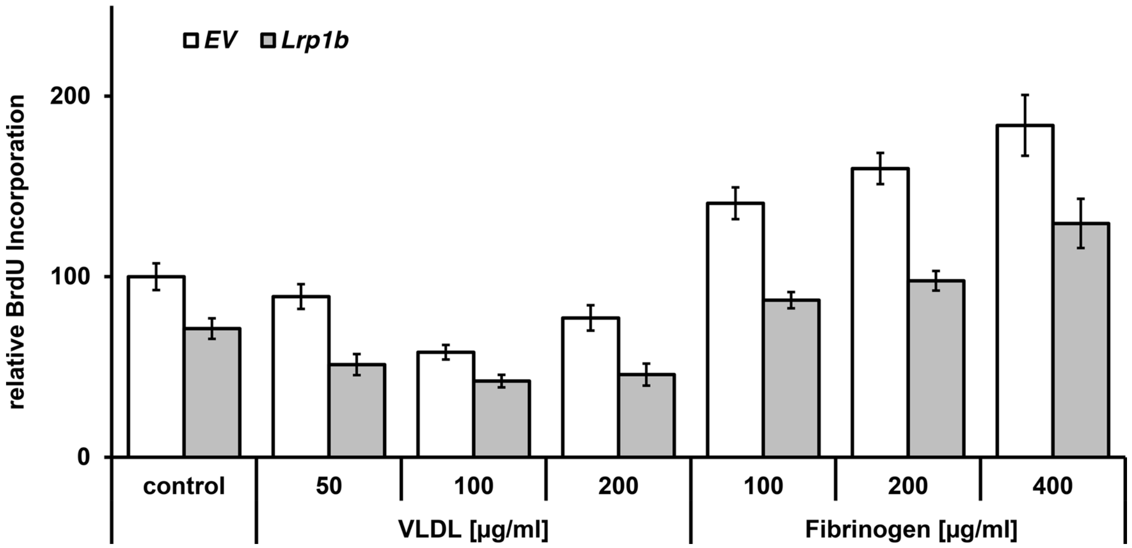

Supplementary Figure S2. Fibrinogen promotes A427 cellular proliferation irrespective of *Lrp1b* overexpression. A427 cells stably transfected with plasmids containing either murine full-length *Lrp1b* or empty vector (EV) control were seeded in 96-well plates at a density of 6 x 103 cells per well in 100 µl culture medium containing 5% fetal calf serum and left to adhere overnight. Thereafter, fresh medium was supplemented with VLDL or fibrinogen at the indicated concentrations. After 48 hours relative quantification of DNA synthesis was determined by a BrdU cell proliferation ELISA according to manufacturer's instructions. Values are given as mean and SEM and are representative for three independent experiments run in quadruplicates. Fibrinogen dose-dependently enhanced proliferation of both *Lrp1b* overexpressing and control cells. Proliferation rate was reduced by 30-40% in A427 cells overexpressing *Lrp1b* irrespective of VLDL or fibrinogen supplementation at the concentrations tested.

Supplemental Table S1: Primer sequences

See Supplementary File 1
