# Supplementary material for: Expression of a recombinant full-length LRP1B receptor in human non-small cell lung cancer cells confirms the postulated growth-suppressing function of this large LDL receptor family member
Source: Oncotarget. 2016 Sep 8;7(42):68721–33. doi: 10.18632/oncotarget.11897 (PMC5356585; doi:10.18632/oncotarget.11897)
Supplement: Supplementary file 2 [file oncotarget-07-68721-s002.docx]

**Supplemental Table 1. Primer sequences.**

| **Cloning Primers** | |
| --- | --- |
|  | **N-Terminal Fragment Primer:** |
| forward | 5´-AATTGCGGCCGCGCCACCATGGCCCAGTTGCTCCTTGCC-3´  (NotI site, Kozak sequence) |
| reverse | 5´-CACTCTCTGACAGCTTGCCCCTGTATATTCTG-3´  (binding site downstream of SfuI site) |
|  | **Middle Fragment Primer:** |
| forward | 5´-CTGATGGGGAAAGCTGCACCAGTG-3´  (binding site upstream of SfuI site) |
| reverse | 5´-TAAGCAAGTTCTATTATCAGCTGCAAGGTAGA-3´  (binding site downstream of EcoRV site) |
|  | **C-Terminal Fragment Primer:** |
| forward | 5´-CCAAGTCACTCAGCCGTGTCCATAAAAC-3´  (binding site upstream of EcoRV site) |
| reverse | 5´-AATTCTTAAGTTATGCTACTGTTTCTCTGATGCCAATTTC-3´  (AflII) |
|  | **Middle Fragment Repair Primer**: |
| forward | 5´-AATTTCCGGAGTTGTTCACATGAAAGTGTATGAC-3´  (BspEI) |
| reverse | 5´-AATTGTTTAAACCCACTTCGGCAACTTCGGTTTTCAC-3´  (PmeI) |
| **Real time PCR Primers** | |
| human *LRP1B* fwd | 5´-CCCCAAAGAGCAGCAAGTCT-3´ |
| human *LRP1B* rev | 5´-CCAAGAGGACGAGAGGCACA-3´ |
| murine *Lrp1b* fwd | 5´- TGGGGGTCTTTTAGAACCAAG-3´ |
| murine *Lrp1b* rev | 5´- TTGTTGGCCCAGAAGTTAGTG-3´ |
| human RPLP0 fwd | 5´- GTCATCCAGCAGGTGTTCGAC-3´ |
| human RPLP0 rev | 5´- CTCCAGGAAGCGAGAATGCAG-3´ |
| **Sequencing Primers** forward | |
| T7 | 5’-TAATACGACTCACTATAGGG-3´ |
| PM67 | 5’-AATTGGATCCTGTGACCCTGGCGAATTTCTTT-3´ |
| PM127 | 5’-CCATAGCAGCAGATCCCATC-3´ |
| PM128 | 5’-AGCGATGGCAGGTCATGCA-3´ |
| PM129 | 5’-GAGAAAGCTTGGATGGATGGT-3´ |
| PM69 | 5’-AATTAGATCTTGTAAACCTGGAGAATTTCGCTG-3´ |
| PM85 | 5’-ATGTTCAAGTGGCCGGTGTATCC-3´ |
| PM130 | 5’-GACTGTGACAGTTTCTTGTGTGGA-3´ |
| PM223 | 5’-GAAAGCTGCACCAGTGTGGA-3´ |
| PM131 | 5’-CCCTCGCATTGAATCTGCTT-3´ |
| PM132 | 5’-AAGCTTTCCTCTGACAAGAAAACC-3´ |
| PM133 | 5’-GGCAAATATGGATGGAAGTAACAG-3´ |
| PM134 | 5’-AAGAACCGCATGTCATGCC-3´ |
| PM135 | 5’-CCCAAGGACTGGATCAACC-3´ |
| PM228 | 5’-TTGCAGTCTTTGGGGCTTAC-3´ |
| PM229 | 5’-AGTACGGCCCTACGAGAACC-3´ |
| PM226 | 5’-TGTTGAGGGCCTCGCCTATC-3´ |
| PM222 | 5’-TCTGAGTTTGAGTGTGGAAATG-3´ |
| PM86 | 5’-TCTGATGAATTAAAGTGCCCAGTTC-3´ |
| PM138 | 5’-CACCCAACAATACATGTGACGA-3´ |
| PM139 | 5’-TCCAACTGAAGGATGATGGTAAA-3´ |
| PM140 | 5’-GATTGGATTGGCAAAAACCTT-3´ |
| PM224 | 5’-CTGATGGGAAAACCAAGTCAC-3´ |
| PM73 | 5’-AATTGGATCCTGCACAGCCAGTCAGTTTCGA-3´ |
| PM141 | 5’-CGGACTGTGCTGATGCCT-3´ |
| PM142 | 5’-GGAGAACCTGACTGTGTTGATGG-3´ |
| PM143 | 5’-GTGATAGCCTTGATGACTGTGGT-3´ |
| PM145 | 5’-CACAGGTCTGACTGTTGATCATTT-3´ |
| PM146 | 5’-ATATATGATGAATGGGACCTGCC-3´ |
| **Sequencing Primers** reverse | |
| BGH rev | 5’-TAGAAGGCACAGTCGAGG-3´ |
| ex89 | 5’-cttctaattgtctttgtt-3´ |
| PM231 | 5’-gcaaattgattccaagagcaag-3´ |
| PM230 | 5’-ctcatagtcaatggagatgcc-3´ |
| PM232 | 5’-cgttctcctgctggttgcat-3´ |
